# Primary subacute talus osteomyelitis caused by *Pasteurella canis*: literature review and case report

**DOI:** 10.1099/acmi.0.000707.v3

**Published:** 2024-01-11

**Authors:** Sohaib Shah, Douglas Donnachie, Noman Niazi, Russell Conyers, Jo Dartnell, Marcos Katchburian, Oluwarantimi Ayodele

**Affiliations:** ^1^​ Department of Trauma and Orthopaedics, Maidstone and Tunbridge Wells NHS Foundation Trust, Pembury, UK

**Keywords:** osteomyelitis of talus, *Pasteurella canis*

## Abstract

In this review, we would like to demonstrate the case of a 6-year-old girl who presented with progressive ankle pain and eventual inability to weight bear. She was shown to have primary acute osteomyelitis of the talus caused by *Pasteurella canis*, a commensal organism usually found in the oropharynx of dogs, despite the absence of any history of a dog bite or other zoonotic risk factors. We characterise the symptoms, signs, radiographic appearances and result of both the medical and surgical management, including a review of the literature. This review aims to increase awareness of this rare pathology and help guide other clinicians in accurately diagnosing and managing the condition.

## Data Summary

All supporting data, code and protocols have been provided within the article.

## Introduction

Osteomyelitis is also known as a bone infection but usually it is caused by infection especially in the arm, legs or spine. It is a serious infection of the bone that can be either acute or chronic that spreads through the bloodstream, nearby areas of infection, fractures, or surgery. Haematogenous osteomyelitis of the talus is a rare cause of ankle pain in a child. Primary subacute osteomyelitis secondary to *Pasteurella canis* infection is reported with the first such case being published in 2002 [1, 2]. However, with both an uncommon and unusual presentation, the diagnosis can often postponed or even overlooked, particularly in the absence of a penetrating dog bite and delayed growth on microbiology specimens. In humans, *P. canis* is known to cause both soft tissue and wound infections, along with systemic bacteraemia. Common diagnoses consist of osteomyelitis, conjunctivitis, peritonitis and arthritis [1].

Subacute osteomyelitis itself is relatively uncommon, with one retrospective review demonstrating it forms around 2.4 % of osteomyelitis cases [3]. On the contrary, in areas such as Eastern Africa, it is reported as the majority of cases [4]. Males seem to have a greater predisposition than females (3 : 2) and appears to affect adolescents with the mean age of 19.5 years [5]. The tibia is the most affected location and is rarely reported in the tarsal bones [4–6].

*Pasteurella* species are native to the oropharynx of healthy Carnivora such as dogs and cats. They are Gram-negative facultative anaerobes which are small and non-motile. *P. multocida* is considered the most common pathogen and species for the genus. Transmission of *P. canis* is often from animals to humans through animal bites, scratches, or licking over wounds [7]. However, there have been rare reports of patients developing *P. canis* infection without any scratches or puncture wounds [1, 8]. Human-to-human transmission has been recorded, including transmission via contaminated blood products and via close contact with colonised individuals [9]. Vertical transmission can occur via genital tract colonisation, transplacental infection or endometritis [8].

The development of subacute osteomyelitis is not clearly appreciated. The diagnosis is established from multiple facets including a thorough history, examination and radiological imaging. However, at initial presentation it is characterised by minimal or no clinical signs and symptoms making early diagnosis extremely difficult [10, 11]. Management remains controversial with treatment including curettage (with or without cancellous bone grafting), biopsies, specimen cultures, the use of antibiotic therapy alone, immobilisation or the use of impregnated antibiotic beads. Regardless, the literature describes benefit from only oral antibiotic treatment [12, 13]. A study by Hamdy *et al*. demonstrated these positive outcomes with administration of antibiotic treatment. The retrospective review compared forty-four cases over twelve years with around half receiving only antibiotics and half initially undergoing surgical debridement with antibiotics post-operatively. There was no conclusive difference between the managements. Therefore, concluding surgical debridement should be utilised in non-responsive cases [14]. This was demonstrated by Olasinde *et al*. where late presentation of subacute osteomyelitis managed with surgical intervention, grafting and antibiotic administration provided excellent results [3]. Collectively, management of subacute osteomyelitis recommends 6 weeks with antibiotic treatment dispensed either completely or in part by an intravenous route [13, 15].

## Case presentation

**Fig. 1.**
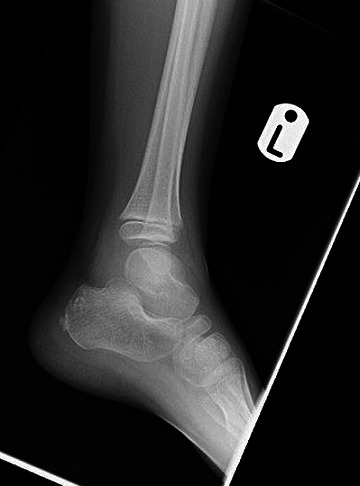
An essentially normal lateral radiograph of the foot demonstrating normal morphology of the talus with no evidence of osteomyelitis, periosteal reaction or evidence of a lytic lesion.

A 6-year-old girl was referred to our Trauma and Orthopaedics Department by our paediatric colleagues with a ten-day history of pain in her left ankle, limping and central abdominal pain. There was no history of animal contact, animal bites or trauma. She was given a possible diagnosis of hand, foot, and mouth disease 3 weeks prior. On presentation, she was systemically well and her inflammatory markers were: C-Reactive Protein (CRP) 1, Erythrocyte Sedimentation Rate (ESR) 89, White Cell Count (WCC) 9.06, Neutrophils 4.11 and normal Rheumatoid Factor (Table 1). On examination, she had a tender swelling and bruising on the anterolateral aspect of her left ankle. She also had full range of motion of both her ankle and subtalar joint. The X-rays were essentially normal (Fig. 1).

**Table 1.** Haematological investigations

	At presentation	Day 4	Day 10	Day 13	Day 15	Day 55
CRP mg l^−1^ (0–5 mg l^−1^)	1	< 1	3	<1	<1	<1
ESR mm hr^−1^ (<15 mm hr^−1^)	89	2	50	22	Not measured	4
Hb g l^−1^ (115–155 g l^−1^)	118	122	115	94	111	119
Neutrophills 10^9^ l^−1^ (1.0–8.0 10^9^ l^−1^)	4.11	5.68	4.72	2.00	4.37	3.28
WBC 10^9^ l^−1^ (4.5–14.5 10^9^ l^−1^)	9.06	10.03	10.33	5.42	10.15	8.19

**Fig. 2.**
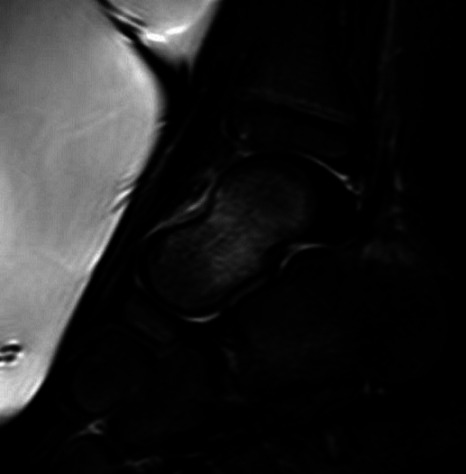
A T2 weighted MRI of the talus. 1) loss of cortical continuity and 2) increased signal in the talus indicating marrow oedema.

She was reviewed by the on-call orthopaedic consultant and the consultant paediatrician and a provisional diagnosis of mesenteric adenitis was made. She was kept under review but no antibiotics were started as she was systemically well and fully weight bearing on her left leg. An outpatient MRI for her left ankle was arranged and she was reviewed on a daily basis as an ambulatory paediatric patient. However, after 1 week, she stopped weight bearing on her left foot completely and developed a low-grade temperature. As a result, she was admitted, and an inpatient MRI was organised. The MRI of her left ankle demonstrated a large joint effusion and a disruption in the cortical outline of the talus in the postero-inferior subtalar facet with surrounding marrow oedema (Fig. 2). Her inflammatory markers, 10 days after the initial blood tests, were CRP 3, ESR 50, WCC 10.33 and Neutrophils 4.72 (Table 1). Her ankle joint was aspirated under ultrasound guidance and she was subsequently started on flucloxacillin. The microscopy revealed Gram-negative rods and after discussion with the microbiology team her antibiotics were changed to co-amoxiclav.

**Fig. 3.**
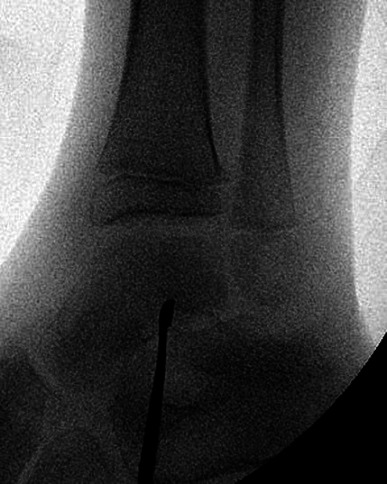
An intra-operative radiograph showing the position of the curette within the sub-talar space.

In view of the positive aspirate and MRI findings, talus osteomyelitis with subtalar septic arthritis was diagnosed. The decision was taken to proceed with drilling and curettage of the talus and washout of the subtalar joint (Fig. 3) with autologous bone grafting.

Operative findings were a 2×1 cm cavity in the body of the talus with 2 ml of pus contained within it. Swabs were taken and sent for microscopy, culture and sensitivity. Unfortunately, at our institution PCR is not available for rapid identification of organisms. Identification and initial anti-microbial sensitivities were performed using a Vitek two machine (produced by BioMérieux). Anti-microbial sensitivities were confirmed by hand using a disc diffusion method on ISO Sensi-Test agar. The aspirate sample taken at ultrasound grew *P. canis*. Her antibiotics were changed, after further discussion on receipt of sensitivities, to ceftriaxone intravenously for 3 weeks followed by 3 weeks of oral amoxicillin (Table 2).

**Table 2.** Antimicrobial susceptibility testing results

Antibiotic	Susceptibility result
Co-trimoxazole	Resistant
Penicillin	Sensitive
Tetracycline	Sensitive
Cefotaxime	Sensitive

## Differential diagnoses

- Septic arthritis of the subtalar joint.
- Talus osteomyelitis.

## Outcome and follow-up

**Fig. 4.**
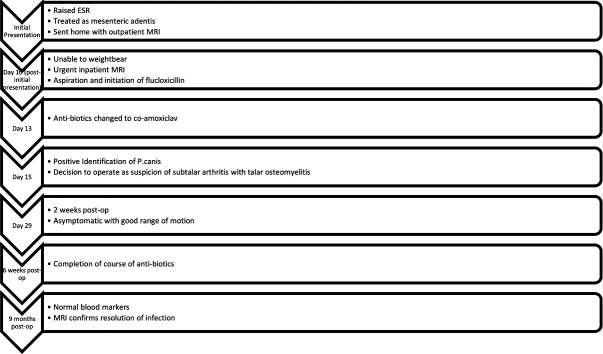
A timeline of events.

**Fig. 5.**
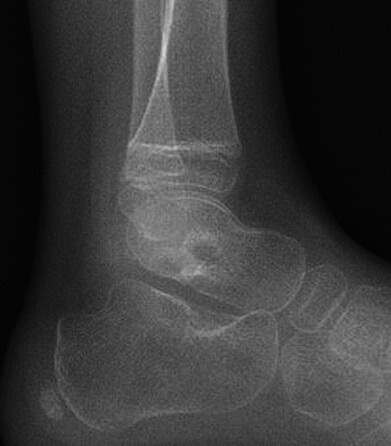
Satisfactory lateral radiograph of the talus showing no signs of osteomyelitis and good bony alignment.

**Fig. 6.**
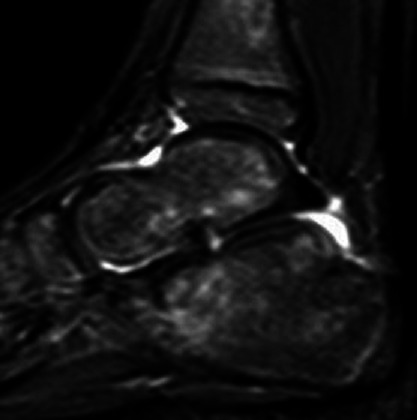
Repeat T2 weight sequence MRI showing resolution of marrow oedema. Cavity left by curettage and filled with bone graft.

The patient improved after both the operation and course of antibiotics (Fig. 4). Two weeks post-operatively she was reviewed and found to be asymptomatic, her wounds had healed sufficiently, she had a good range of motion in her ankle, normal bloods and a normal X-ray (Fig. 5). She was still asymptomatic at 4 weeks at which point weight bearing was started. She is still under follow up 9 months post-op with no recurrence of her symptoms and a repeat MRI confirmed complete resolution of infection (Fig. 6).

## Discussion

The reported incidence of osteomyelitis varies from 1 to 13 per 0.1 million population in developed nations, with higher values of up to 200 per 0.1 million population reported in developing countries [16]. Isolated involvement of the talus is rare with a minority of cases reported [17]. Primary osteomyelitis of the tarsal bones in children is rare but is a definite clinical syndrome which may pose diagnostic conundrums secondary to the absence of both clinical and laboratory findings related to bone infection [11]. The utility of laboratory investigations in paediatric osteomyelitis has previously been examined in a systematic review by Dartnell *et al*. It highlighted that ESR and CRP are by far the most sensitive markers, with derangements in their values 91 and 80.5 % of the time respectively [18]. However, in this case, although the ESR was markedly raised, the CRP was normal. In conjunction with the longer half-life of a raised ESR and the recent history of possible hand, foot, and mouth disease, this made the blood investigations even more difficult to interpret. In such an insidious pathology, laboratory tests may therefore be misleading.

Indeed, clinical features may prove to be more helpful. Dartnell *et al*. revealed that 80 % of paediatric osteomyelitis cases present with pain and 70 % with localising signs, as demonstrated in this case [18]. Unfortunately, these findings are often non-specific, and this illustrates the diagnostic challenge faced.

In terms of the proposed pathophysiology, Nixon describes flat and irregular bones, including the talus, as having anatomic subdivisions comparable to those of long bones. He termed these subdivisions metaphyseal-equivalent locations because they have a vascular anatomy similar to that of metaphysis [17]. This similarity of vascular anatomy may predispose them to developing osteomyelitis via haematogenous spread [18]. Just as the metaphysis are commonly involved in haematogenous osteomyelitis, so are these metaphyseal-equivalent locations in flat and irregular bones affected. These areas lie adjacent to cartilage and border apophyseal growth plates, articular cartilage, or fibrocartilage. This theoretical underpinning of the development of talar osteomyelitis is currently unproven as it remains both an uncommon location and rare presentation. This rarity may preclude correct recognition of subacute osteomyelitis of the talus bone and cause unwanted waits, something we hope to ameliorate with this case report.

The haematological process of spreading is similar to other infectious disease such as tuberculosis. Previously, Khan *et al*. suggested that osteoarticular tuberculosis (OATB) belongs to the extrapulmonary TB (EPTB) category that may lead to osteomyelitis due to considerable delay in diagnosis [19]. Thus retrograde lymphatic and contiguous dissemination are lesser modes of transmission and OATB normally begins as osteomyelitis in the growth plates of bones and is transmitted into the joint spaces. Spinal TB affects mainly dorsal and lumbar vertebrae, which if not diagnosed/treated correctly can develop into kyphosis and/or permanent neurological damage [20].

Presentation can be sudden onset but with little evidence on history, examination and the patient feeling remarkably well. Unfortunately, rendering challenges to recognise, subsequently leading to possible waits in implementing definitive therapy. Findings can vary but the typical radiological features of the disease on MRI are a lytic cavity with sclerotic margins. Important differentials are intraosseous bone cyst, osteoid osteoma, aneurysmal bone cyst, chondroblastoma and lipoma. In many instances, biochemical markers including CRP, ESR, WCC and blood cultures often provide no additional benefit. As demonstrated in this case presentation, the infective diagnosis was not fully backed by biochemical markers.

A systematic review in 2012 suggested guidelines, of which appropriate anti-microbial therapy is the mainstay with surgical intervention being reserved for cases with a lack of clinical improvement and persistently deranged laboratory results [21]. For reasons previously discussed, laboratory investigations may not always be of use. The case presented reasoning for surgical intervention with curettage and bone grafting was indicated based on the clinical deterioration, inability to weight bear and reduced range of motion in her subtalar joint, likely due to secondary septic arthritis of the subtalar joint. While the positive aspirate results and MRI findings helped the diagnosis, the unusual nature of the infection with a lack of literature around its treatment also made the case for a more aggressive approach. The fact that there was no clinical improvement despite 5 days of appropriate therapy meant that, in our opinion, source control was necessary. Having completed the aforementioned regimen of anti-microbials and operative intervention, the follow-up outcome at 9 months was satisfactory both radiologically and clinically.

## Learning points

- Bone and joint infections secondary to *Pasteurella* spp. are infrequently reported and a rare occurrence in paediatric literature.
- The potential for significant morbidity and mortality associated with deep tissue infections due to *Pasteurella* spp. places emphasis on the significance of surgical debridement, appropriate antibiotics and wound care.
- Early surgical debridement of joint spaces and appropriate antibiotics can reduce morbidity and long-term complications.
- Surgical treatment of acute osteomyelitis with collection in the weight bearing joints is important for long term prognosis.
